# Colon Single Stripe Sign in Chronic NSAID Use During Norovirus Infection: A Two‐Hit Model of Ischemic Colitis

**DOI:** 10.1002/ccr3.73524

**Published:** 2026-09-15

**Authors:** Sushrut Ingawale, Rahul Kothari, Pujitha Vallivedu, Shivaji Karki, Gagandeep Singh Talwar, Jack Jacob, David Regelmann, Prabin Sharma

**Affiliations:** ^1^ Frank H. Netter MD School of Medicine Quinnipiac University, St. Vincent's Medical Center Bridgeport Connecticut USA; ^2^ Seth Gordhandas Sunderdas Medical College and King Edward VII Memorial Hospital Mumbai Maharashtra India; ^3^ Institute of Medicine, Tribhuvan University Teaching Hospital Kathmandu Nepal; ^4^ Montgomery Family Medicine Residency Program, Baptist Health Partners Montgomery Alabama USA

**Keywords:** abdominal pain, acute kidney injury, colonoscopy, diarrhea, drug‐related side effects and adverse reactions, gastrointestinal hemorrhage, ischemic colitis, non‐steroidal anti‐inflammatory agents, norovirus

## Abstract

The colon single stripe sign (CSSS) is associated with ischemic‐pattern colonic injury. We report a 69‐year‐old woman with norovirus infection, hematochezia, recurrent left‐sided colitis, and chronic NSAID use found to have CSSS on colonoscopy, suggesting a multifactorial “two‐hit” mechanism involving NSAID‐related mucosal vulnerability and transient hypoperfusion.

## Key Clinical Message

Recognition of the colon single stripe sign should prompt consideration of ischemic‐pattern colonic injury, particularly in patients with chronic NSAID exposure and superimposed dehydration or infection. This case highlights a possible “two‐hit” mechanism linking chronic NSAID use and transient infectious insults to ischemic colitis.

**Abbreviations**
AKI: acute kidney injury
CSSS: colonic single stripe sign
EPEC: Enteropathogenic *E. coli*

IC: ischemic colitis
ICAM‐1: Intercellular Adhesion Molecule 1
NSAID: nonsteroidal anti‐inflammatory drug
PCR: polymerase chain reaction
TNF‐α: tumor necrosis factor‐alpha

## 1 Introduction

The “colon single stripe sign” (CSSS) was first described in a study published in 2003 by Zuckerman et al. [1], where 26 patients exhibiting the CSSS were retrospectively studied. The CSSS refers to the presence of a single inflammatory band of erythema, erosions, and ulceration, along the longitudinal intestinal axis without circumferential involvement [1]. The sign is seen mostly on the left colon. The presence of CSSS was associated with 62% of patients exhibiting evidence of a preceding ischemic event, and 75% of histopathological examinations revealing microscopic evidence of ischemic injury. The patients with CSSS had lower mortality [1]. Subsequent studies have suggested that the presence of CSSS may represent a milder or transient form of ischemic injury, often associated with favorable outcomes. Although initially described in association with ischemic colitis (IC), the CSSS is not pathognomonic and should be interpreted in the appropriate clinical context, particularly in patients with overlapping infectious or drug‐related risk factors.

IC is characterized by a state of vascular compromise to the large intestine, resulting in an inability to meet metabolic demand. IC classically presents with rapid onset of abdominal pain, tenderness, and diarrhea or hematochezia, typically with acute onset. IC may be seen in the context of systemic hypoperfusion, hypercoagulability, vascular disease, or iatrogenic drug administration [2]. On colonoscopy, IC has been found to be associated with the CSSS [1]. However, mild IC may often not be diagnosed, due to being misdiagnosed or not reported owing to its transient presentation, especially in mild cases [2]. Furthermore, CSSS, even if present, may often not be accorded its due significance [1].

We present a case of chronic naproxen use presenting with chronic intermittent lower abdominal pain and found to have CSSS during a Norovirus outbreak. A further review of the literature into similar published cases reveals a possible association with chronic nonsteroidal anti‐inflammatory drug (NSAID) use.

## 2 Case History and Examination

A 69‐year‐old white woman with a history of partial thyroidectomy, type 2 diabetes mellitus, hypertension, hyperlipidemia, mild mitral stenosis, spinal stenosis managed surgically, and patent foramen ovale presented to the emergency department with lower abdominal pain and diarrhea for 5 days. She had a history of chronic intermittent lower abdominal pain and diarrhea for around 1 year. She also had a hospitalization for colitis, with imaging revealing non‐specific colitis and stool cultures confirming the presence of Enteropathogenic *E. coli* (EPEC), treated optimally with cephalosporins.

At the current presentation, the pain was described as constant and sharp. Its onset was initially associated with diarrhea, nausea, vomiting, and chills, though these symptoms resolved. The diarrhea persisted intermittently with the pain. She had an episode of bloody diarrhea in the emergency department. She denied experiencing bloody stool previously, melena, dysuria, hematuria, or fevers. The patient's last colonoscopy, more than 10 years ago, had been normal. On examination, the patient was afebrile, with a heart rate of 70 beats per minute, blood pressure 99/67 mmHg, and normal oxygen saturation on room air. There was mild abdominal discomfort to palpation in the lower quadrants.

## 3 Differential Diagnosis, Investigations, and Treatment

The differential diagnoses for this patient's recurrent lower abdominal pain, diarrhea, hematochezia, and left‐sided colitis are described in Table 1.

**TABLE 1:** Differential diagnosis of recurrent left‐sided colitis and hematochezia.

Diagnosis	Points favoring diagnosis	Points disfavoring diagnosis
Ischemic colitis	Advanced age and multiple vascular risk factors (hypertension, diabetes mellitus, hyperlipidemia); recurrent left‐sided colonic involvement in watershed regions (splenic flexure, descending and sigmoid colon); hematochezia; colonoscopic finding of the characteristic colon single‐stripe sign (CSSS); histology showing ulceration with features of chronicity consistent with ischemic‐pattern injury; chronic NSAID use increasing susceptibility to ischemic injury; clinical improvement with supportive care and NSAID cessation	Histopathology was not entirely specific for ischemia and lacked definitive transmural ischemic changes
Infectious colitis	Prior hospitalization with Enteropathogenic *E. coli* (EPEC) colitis; positive stool PCR for norovirus during a regional outbreak; acute onset with diarrhea, nausea, vomiting, and chills; CT findings consistent with colitis	Recurrent and localized left‐sided distribution across multiple imaging studies was atypical for infectious colitis; persistence of hematochezia despite supportive care; absence of fever, leukocytosis, or systemic inflammatory response syndrome; endoscopic CSSS favored ischemic rather than infectious injury
Inflammatory bowel disease (particularly ulcerative colitis)	Chronic intermittent abdominal pain and diarrhea for approximately 1 year; hematochezia; biopsy showing active colitis with focal chronic features	Lack of prior inflammatory bowel disease history; absence of diffuse continuous colitis or classic endoscopic findings of ulcerative colitis; no systemic inflammatory features; older age at presentation made new‐onset inflammatory bowel disease less likely; symptom resolution without immunosuppressive therapy
NSAID‐induced colopathy	Daily long‐term NSAID use (naproxen and meloxicam) for over 3 years; NSAIDs are associated with mucosal ulceration, colitis, and ischemic susceptibility; chronic and recurrent symptoms	NSAID‐induced injury alone did not fully explain the characteristic ischemic distribution and CSSS; histology and endoscopic findings more strongly supported ischemic‐pattern injury
Diverticular‐associated colitis	Presence of left‐sided diverticulosis; symptoms of abdominal pain and hematochezia	Endoscopic findings were more characteristic of ischemic colitis; inflammation extended beyond areas typically affected by diverticular‐associated colitis; histology lacked features specific for segmental colitis associated with diverticulosis
Acute mesenteric ischemia	Abdominal pain and hematochezia in an elderly patient with vascular risk factors	Hemodynamically stable presentation without severe disproportionate pain; absence of peritoneal signs, lactic acidosis, or systemic toxicity; colonoscopy and imaging findings were more consistent with localized ischemic colitis rather than acute transmural mesenteric ischemia

**FIGURE 1:**
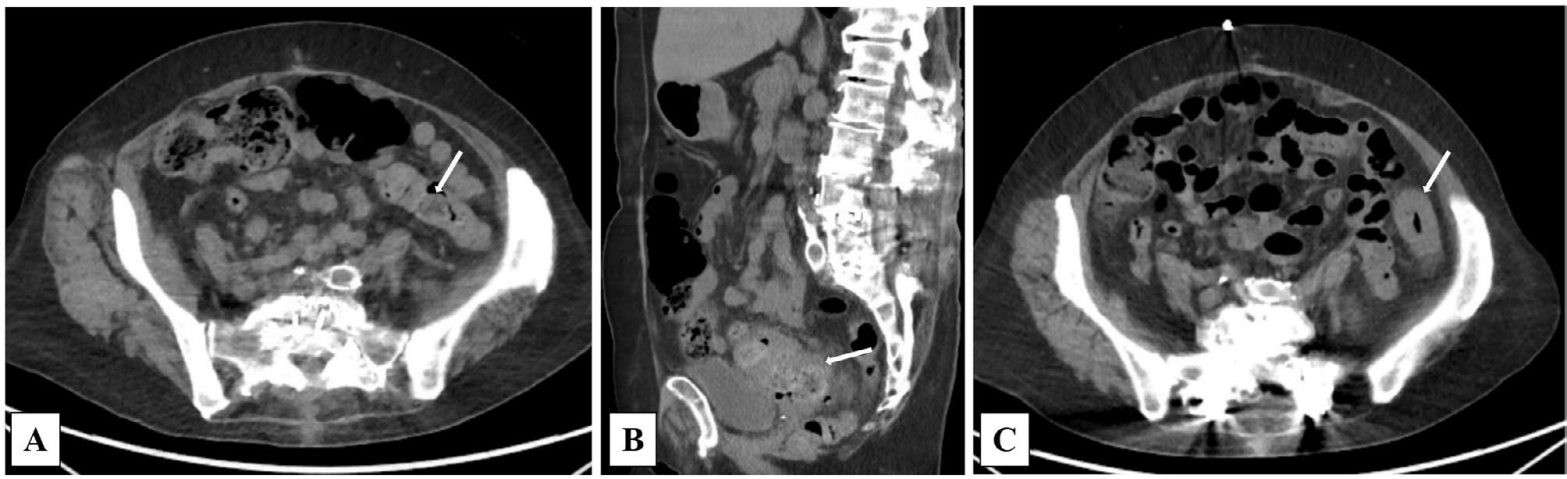
Non‐contrast CT of the abdomen and pelvis. (A) Transverse and (B) sagittal views obtained at the current presentation, and (C) transverse view obtained 5 months prior, demonstrating persistent mild wall thickening (white arrows) involving the distal transverse, descending, and sigmoid colon, with subtle adjacent pericolic fat stranding, findings suggestive of colitis. No lymphadenopathy is identified. Multiple colonic diverticula are incidentally noted without evidence of acute diverticulitis. Atheromatous calcifications of the abdominal aorta and its branches are also noted, without CT evidence of vascular occlusion or ischemic bowel.

Laboratory evaluation showed normocytic anemia (hemoglobin 10.3 g/dL) and elevated creatinine (3.2 mg/dL) with mild hyperkalemia (5.1 mmol/L) due to acute kidney injury (AKI), likely in the setting of diarrhea‐induced volume depletion. But there was no leukocytosis or other signs of Systemic Inflammatory Response Syndrome. A computed tomography (CT) scan of the abdomen and pelvis without contrast showed mild thickening of the distal transverse, descending, and sigmoid colon with pericolonic fat stranding, suggestive of colitis as seen in Figure 1. This was similar to the CT scan 5 months during her last hospitalization, which reported diffuse thickening of the colon, particularly from the splenic flexure, descending, sigmoid colon, and rectum with loss of haustrations. A stool polymerase chain reaction (PCR) panel was positive for norovirus. At the time, there was an outbreak of norovirus in the state of Connecticut.

**FIGURE 2:**
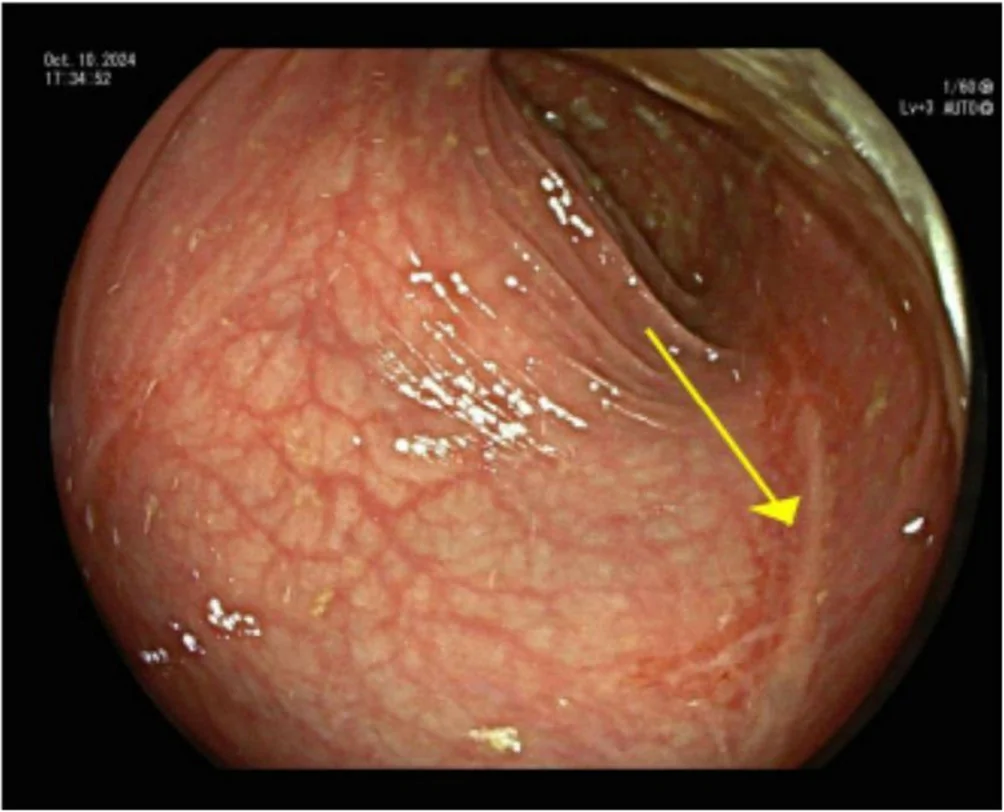
Colon single stripe sign (CSSS) identified on colonoscopy: The colonscopy demonstrates a longitudinal band (yellow arrow) in the left colon (50 cm), without circumferential involvement.

**FIGURE 3:**
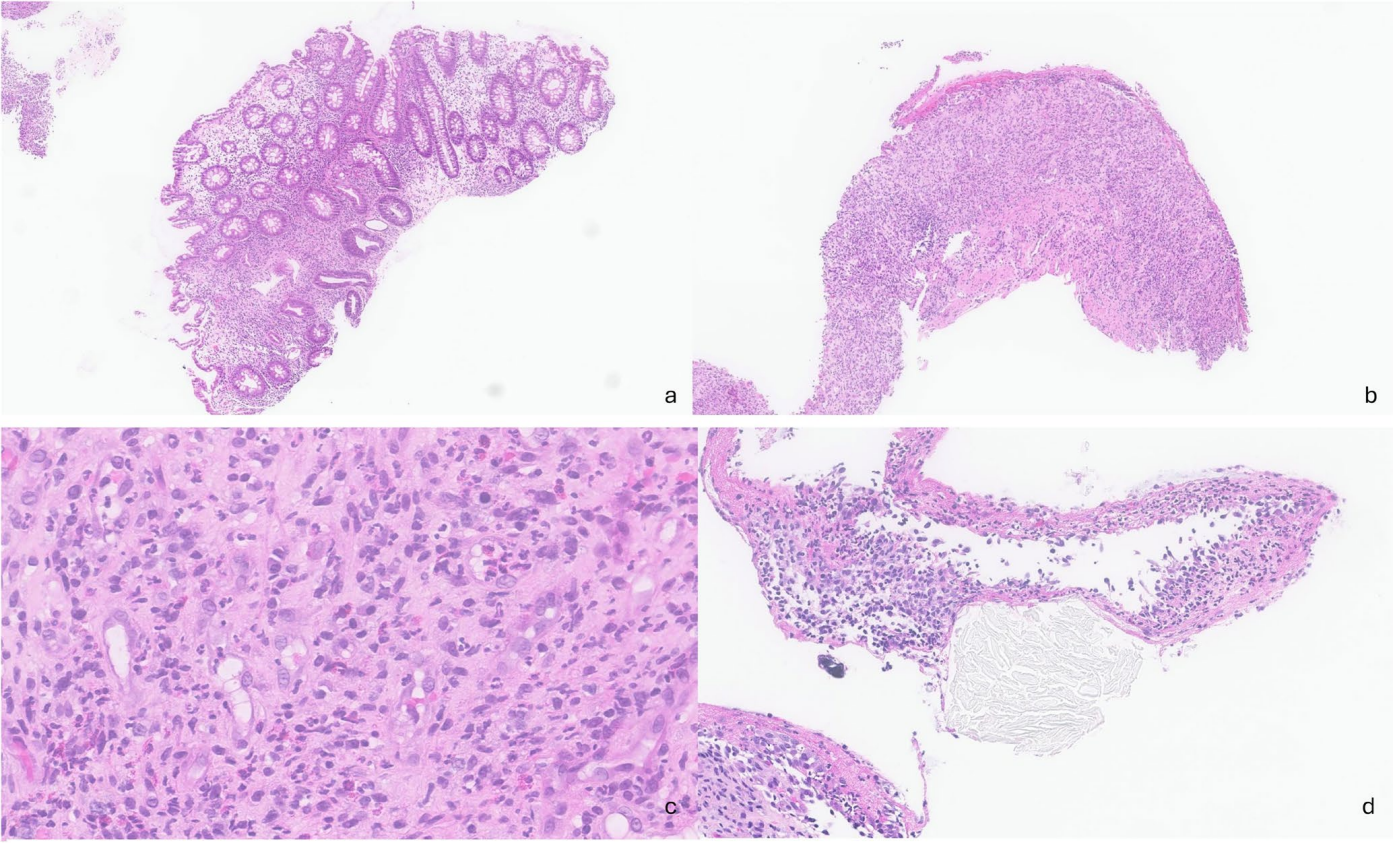
Histopathologic features of colonic biopsies obtained from the affected segment. (a) Low‐power view of colonic mucosa from 35 cm proximal to the anus showing active colitis without significant chronic architectural distortion. (b) Low‐power view of a separate tissue fragment demonstrating ulcerated colonic mucosa with associated granulation tissue. (c) High‐power view highlighting granulation tissue with acute inflammatory infiltrate, including eosinophils. (d) Ulcerated mucosa with overlying inorganic foreign material (was negative for iron staining).

She was treated with intravenous fluids, morphine for pain control, supportive measures, and isolation. Her AKI resolved. However, the hematochezia persisted with a drop in hemoglobin up to 8.7 g/dL. She underwent a colonoscopy. There was no evidence of any active bleed. But it showed erythematous mucosa 35–50 cm proximal from the anus with the CSSS at 50 cm, as seen in Figure 2. Diverticulosis was also observed in the left colon. Biopsies were taken. Microscopic examination showed ulcerated colonic mucosa with active colitis and focal features suggestive of chronicity, as seen in Figure 3. While histologic findings were not specific for IC, the presence of mucosal ulceration with features of chronicity, in conjunction with the characteristic endoscopic appearance and clinical presentation, supported an ischemic‐pattern injury rather than isolated infectious colitis.

The recurrent left‐sided distribution of colonic involvement across imaging studies raised concern for a localized vulnerability rather than diffuse infectious colitis. On further inquiry, the patient reported daily NSAID use for over 3 years, including naproxen 500 mg and meloxicam 15 mg, raising concern for chronic NSAID‐associated colonic injury and ischemic susceptibility.

## 4 Outcome and Follow‐Up

Patient clinically improved. The patient's hemoglobin remained stable. She was discharged home. On subsequent outpatient follow‐up, the patient reported complete resolution of her clinical symptoms. The patient was advised to limit the use of NSAIDs.

## 5 Discussion

IC is the most common form of intestinal ischemia and results from a transient or sustained reduction in colonic perfusion. It classically presents with acute‐onset abdominal pain, diarrhea, and hematochezia, particularly in older adults with vascular risk factors. Colonoscopy remains a key diagnostic tool, and among its characteristic findings, the CSSS has been described as a longitudinal band of erythema, erosion, or ulceration, most often involving the left colon. While not pathognomonic, CSSS has been associated with milder ischemic injury and favorable clinical outcomes [1].

In this case, the patient presented with clinical features compatible with IC, including abdominal pain, hematochezia, hypotension, and left‐sided colitis on imaging. Colonoscopy revealed CSSS with histologic evidence of active colitis and focal chronic changes. The diagnostic challenge lies in the coexistence of a confirmed norovirus infection, which may independently cause colitis, volume depletion, and transient hypotension. However, norovirus typically produces diffuse mucosal inflammation rather than a focal longitudinal lesion, and the recurrent left‐sided distribution of disease in this patient suggests an underlying susceptibility.

Chronic NSAID use is a compelling contributory factor. NSAIDs are well known to cause gastrointestinal injury beyond the upper tract, with colonic manifestations including diaphragm‐like strictures, ulceration, exacerbation of inflammatory bowel disease, and ischemic‐pattern injury. Proposed mechanisms include inhibition of prostaglandin‐mediated mucosal protection, microvascular vasoconstriction, mitochondrial dysfunction, topical epithelial toxicity, and upregulation of inflammatory mediators such as Tumor Necrosis Factor‐alpha (TNF‐α) and Intercellular Adhesion Molecule 1 (ICAM‐1). A few reports have documented NSAID‐associated IC in patients without overt vascular disease, suggesting that NSAIDs may precipitate ischemia even in the absence of major arterial occlusion [3, 4, 5]. The mechanism for NSAID‐induced IC has been hypothesized to involve local microvascular spasm triggered by prostaglandin synthesis inhibition, uncoupled mitochondrial phosphorylation, topical toxicity, and increased expression of TNF‐α and ICAM‐1 [3].

In the present case, chronic NSAID exposure likely created a baseline state of mucosal vulnerability. This may have been unmasked or exacerbated by acute volume depletion and hypotension related to norovirus infection, resulting in a “two‐hit” phenomenon. Additional patient‐specific factors, including advanced age, diabetes mellitus, mitral stenosis, and transient renal dysfunction, may have further impaired colonic perfusion. Importantly, the presence of CSSS supports an ischemic injury pattern rather than primary infectious colitis alone.

A review of the literature regarding NSAID‐associated colonic injury suggests that IC is just one of the manifestations in the spectrum of possible lesions [6, 7, 8]. The exact manifestations may depend on the dosage and chronicity of usage, and may be modified by the presence of other disease processes. Since management may vary depending on the pathology observed, the presence of the CSSS becomes important. Table 2 summarizes the published cases of CSSS, but they had varied etiologies for colitis [9, 10, 11, 12, 13, 14, 15, 16].

**TABLE 2:** Summary of previously reported cases with CSSS.

Report	Demographics	Comorbidities	Medications	Clinical presentation	Attributed etiology
Current (Ingawale et al. 2025)	69, Female	Partial thyroidectomy, hypertension, hyperlipidemia, diabetes mellitus, mitral stenosis, patent foramen ovale, spinal stenosis, infective colitis	Naproxen, meloxicam, oxycodone	Abdominal pain, tenderness, diarrhea, bloody stool, nausea, vomiting, chills	Unclear, potential role of NSAIDs
Badar 2024 [9]^a^	87, Male	Prostate cancer (on radiotherapy)	—	Abdominal pain, diarrhea	Not specified
Nagi 2024 [10]	48, Female	Grave's disease, hypertension	Phentermine, valsartan	Syncopal episode, abdominal pain, bloody diarrhea	Chronic phentermine use
Sotiropoulos 2024 [11]^a^	79, Male	COPD	—	Melena	Not specified
Bouvette 2023 [12]^a^	80, Male	Atrial fibrillation, hypertension, repaired aortic aneurysm	—	Hematochezia	Atrial fibrillation in the setting of atherosclerosis
Parikh 2019 [13]	62, Female	Tobacco use, hypertension, constipation, chronic headaches	Ibuprofen	Abdominal pain, bleeding per rectum	Not specified (Possibly NSAID use)
Anderson 2017 [14]	64, Female	Hypertension, cervical cancer, colostomy	—	Bleeding through colostomy	Radiation exposure, hypotension
Tanapanpanit 2015 [15]	81, Female	Not specified	—	Abdominal pain, bloody diarrhea	Not specified
Hogenauer 2008 [16]	62, Female	Munchhausen syndrome	—	Chronic gastrointestinal bleeding	NSAID use

^a^ Published conference abstracts or posters.

The recognition of CSSS in this context has important clinical implications. Mild or transient IC is frequently underdiagnosed or misattributed to infectious etiologies, particularly when symptoms resolve with supportive care. Identifying CSSS should prompt clinicians to evaluate reversible risk factors, including NSAID use, and to consider medication discontinuation to prevent recurrence or progression.

## 6 Conclusion

In conclusion, this case highlights the importance of recognizing the CSSS as a marker of ischemic‐pattern colonic injury within the broader spectrum of NSAID‐associated disease. Greater awareness of this association may facilitate earlier diagnosis, targeted management, and improved outcomes.

## Author Contributions

**Sushrut Ingawale:** conceptualization, investigation, validation, formal analysis, supervision, writing – original draft, writing – review and editing, resources. **Rahul Kothari:** investigation, formal analysis, writing – original draft, resources. **Pujitha Vallivedu:** investigation, writing – original draft, validation. **Shivaji Karki:** investigation, validation, formal analysis, writing – review and editing, writing – original draft, supervision, conceptualization. **Gagandeep Singh Talwar:** investigation, validation, writing – original draft. **Jack Jacob:** investigation, formal analysis, validation, writing – review and editing, resources. **David Regelmann:** writing – review and editing, investigation, validation. **Prabin Sharma:** conceptualization, investigation, formal analysis, writing – review and editing.

## Funding

The authors have nothing to report.

## Consent

Written informed consent was obtained from the patient for publication of this case report and accompanying images.

## Conflicts of Interest

The authors declare no conflicts of interest.

## Data Availability

The de‐identified clinical data supporting the findings of this case report are available upon reasonable request, subject to author approval to protect patient confidentiality.
